# Consumer awareness, purchase behavior, and brand loyalty in local and regional dairy markets

**DOI:** 10.3168/jdsc.2026-1127

**Published:** 2026-10-05

**Authors:** Pralhad Bajgain, Alicia Rihn, Elizabeth Eckelkamp

**Affiliations:** Departments of Agricultural and Resource Economics and Animal Sciences, University of Tennessee, Knoxville, TN 37996

## Abstract

This study examined consumer responses to local and regional dairy brands in Tennessee, including their progression through a 4-stage marketing funnel (brand awareness, purchase intent, repurchase likelihood, and recommendation likelihood) when considering 7 dairy brands (6 real, 1 hypothetical). Using an in-person survey, we found that brand awareness represented the binding constraint for local small creameries, with brand awareness rates below 10%, whereas awareness was 93% for the established regional brand. Engagement with farm-direct retail channels and approval of local food buying promoted local dairy brand awareness and initial purchase likelihood for those products. Conditional on purchase, perceived quality emerged as the dominant driver of both repurchase and recommendation intentions. The local brand exhibited significantly higher recommendation likelihood than purchasers of the regional brand, suggesting word-of-mouth advocacy among engaged consumers. Closing the awareness gap represents the major challenge for regional and local dairy brands.

## Graphical Abstract

**Figure:**
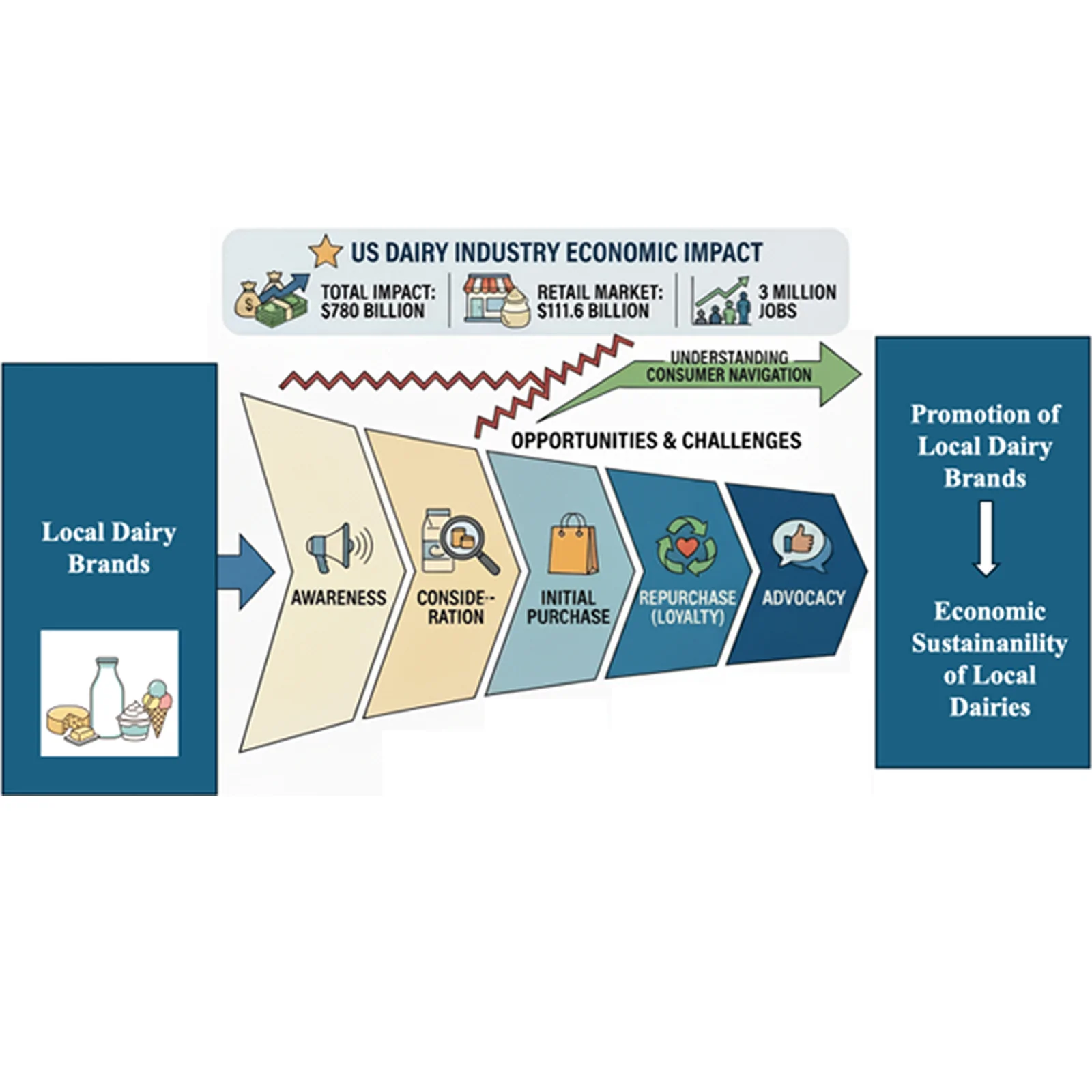

The dairy industry occupies a central position in the US food system. According to the International Dairy Foods Association, the industry's 2025 total economic impact is nearly $780 billion, collectively representing a retail market valued at $111.6 billion, and it creates $198 billion in direct wages with 3 million jobs (IDFA, 2021). Despite consolidation in production and processing (MacDonald et al., 2020), recent years have witnessed a resurgence of interest in locally and regionally produced dairy products (Printezis and Grebitus, 2018; Bajgain et al., 2026), driven by shifting consumer values and local food's association with environmental sustainability (Coelho et al., 2018; Varziri et al., 2025) and support for rural livelihoods (Rossi and Woods, 2024).

Consumer preferences for local dairy products present opportunities and challenges. Small- and medium-scale farm-direct dairies can tap into the premium market segments (Esene, 2023; Bajgain et a., 2026), yet they face barriers related to limited brand visibility (Zaring et al., 2024), consumer awareness, and the capacity to convert awareness to sustained purchasing loyalty (Zaring et al., 2024). Understanding how consumers navigate from initial brand awareness to purchase loyalty and ultimately brand advocacy (i.e., recommendations) is a fundamental question in agricultural marketing research. This marketing funnel approach follows Li and Kannan (2014) and de Haan et al. (2016), which provide a stepwise structural lens to examine these components of consumer decisions. The funnel conceptualizes consumer engagement across different stages: brand awareness, purchase consideration, initial purchase, repurchase, and recommendation to others. Understanding what factors influence these conversions is important when developing efficient marketing strategies (Lemon and Verhoef, 2016), including for local dairy producers.

The local food movement has been extensively documented in the literature (DeLind, 2011; Printezis and Grebitus, 2018). Studies demonstrate that consumers who value local food express higher willingness to pay (**WTP**) premiums for local or regional products (Printezis et al., 2019). However, stated WTP is influenced by many factors, such as limited product availability and brand recognition (Kristensen et al., 2012). For local dairy brands, consumer awareness remains a critical constraint even when local products are available in retail channels, because many consumers are simply unaware of them (Bradford and Durst, 2021). Lack of awareness limits consumers' ability to make informed purchasing decisions. For local dairy producers, who typically have lower advertising budgets than larger national brands, building awareness is particularly challenging. Research has shown that perceptions and attitudes toward local foods play a larger role in local food purchase decisions than demographic characteristics (Zepeda and Li, 2006; Cranfield et al., 2012). Moreover, prior participation in farm-direct purchasing channels (e.g., farmers markets, community-supported agriculture [**CSA**] programs) positively influence consumer perceptions of local foods and brand familiarity (Printezis et al., 2019). Beyond awareness, the transition to actual purchase is shaped by both brand-level and consumer-level characteristics. Perceived product quality is a primary driver of dairy purchase decisions (Esene, 2023). Consumers who associate local or farm-direct dairy products with higher quality are more likely to make initial purchases and remain loyal. Brand recommendation is among the most important, cost-effective, and efficient marketing mechanisms available to small producers who lack resources for mass-media advertising (Hogan et al., 2004).

Despite its importance, the determinants of both repurchase and recommendation likelihood for local dairy brands while controlling for brand heterogeneity have not been assessed in the literature. This study addresses this gap by using a consumer survey conducted in a regional US dairy market, Tennessee, to examine the 4-stage marketing funnel in the context of local and regional dairy brands. The 4 stages include brand awareness, purchase likelihood (conditional on awareness), repurchase likelihood (conditional on prior purchase), and recommendation likelihood (conditional on prior purchase). Six dairy brands were identified, ranging from a large established regional brand to several regional creameries (local brands A, B, C, D, and E; **LA**, **LB**, **LC**, **LD**, and **LE**, respectively). Brands were selected based on local or regional brands in Tennessee, from hyperlocal producers and East Tennessee regional providers to broader intrastate Middle Tennessee farmsteads. The retail channels of these farms vary from mass retail to on-site shops, wholesale stores, select specialty grocers, and CSA farm boxes. Additionally, a hypothetical brand was developed using the Google Gemini Nano Banana artificial intelligence image generator, using keywords “logo,” “dairy creamery,” and “name” (https://gemini.google.com/). The brands were included to allow both brand-level heterogeneity and consumer-level predictors in the progression of the funnel. The specific study objectives were (1) to characterize consumer awareness of local dairy brands to a larger regionally established brand; (2) to quantify funnel conversion rates across the awareness-purchase-repurchase-recommendation pathway; (3) to identify the consumer-level, attitudinal, and brand-level factors that predict progression at each stage of the marketing funnel; and (4) to derive policy implications for marketing strategies among local dairy producers seeking to expand their market penetration.

To preserve brand anonymity and investigate our primary research question (i.e., consumer responses to local versus regional brands), detailed brand-level information was not explicitly controlled for in our regression. Although apparently ignored in our analysis at first glance, these secondary attributes inherently characterize and reinforce the distinction between regional and local farmstead dairies.

Primary data were collected through a structured in-lab consumer survey hosted in Qualtrics at the University of Tennessee (Knoxville, TN) in the summer of 2024. Respondents were adults and purchasers of dairy products. The survey received institutional review board approval for human subjects research (IRB-24-08051-XM). The questionnaire addressed (1) household dairy purchasing habits, including purchase frequency across product categories and expenditures; (2) retail channels used; (3) brand awareness and purchase for 7 brands; (4) repurchase and recommendation likelihood for previously purchased brands, and (5) sociodemographic characteristics. The sample comprised 110 respondents, and each contributed to the brand-level observations across brands, yielding a pooled data set suitable for panel-style analysis. To identify the consumer-level and brand-level factors associated with progression at each stage of the funnel, a series of linear regression models were estimated. Each model incorporated the brand-level controls, with SE clustered at the respondent level. This approach follows recommendations for multilevel consumer survey data where observations are nested within respondents who each evaluate multiple brands, inducing within-respondent correlation in errors (Cameron and Miller, 2015). Because each respondent responded to the different brands' awareness, we restructured the data such that each respondent-brand pair became 1 observation. This was necessary to estimate a single model with brand-level controls rather than running separate models for each brand. For model 1, the dependent variable was brand awareness (1 = aware; 0 = otherwise). Model 2 addressed purchase behavior (1 = purchased before; 0 = otherwise). Thus model 1 and 2 are linear probability models. If participants had purchased the brand previously, they were asked about repurchase (model 3) and recommendation likelihoods (model 4). In models 3 and 4, the dependent variables were repurchase likelihood and recommendation likelihood, using a continuous scale from 0 to 100, where 0 = “0% not at all likely” and 100 = “100% likely to” repurchase or recommend. Additional attitudinal and demographic characteristics were incorporated into each model. Specifically,

[1] $$\text{\textbackslash{}[}\begin{array}{c} Aware_{ib}=\\ \alpha +\beta_{1}NoFarmDirectKnowledge_{i}+\beta_{2}FarmDirectPF_{i}\\ +\beta_{3}DairySpending_{i}+\beta_{4}LocalApproval_{i}+X_{i}\gamma +\delta_{b}+\varepsilon_{ib}, \end{array}\text{\textbackslash{}]}$$

[2] $$\text{\textbackslash{}[}\begin{array}{c} Purchase\;likelihood_{ib}=\\ \alpha +\beta_{1}NoFarmDirectKnowledge_{i}+\beta_{2}FarmDirectPF_{i}\\ +\beta_{3}DairySpending_{i}+\beta_{4}LocalApproval_{i}+X_{i}\gamma +\delta_{b}+\varepsilon_{ib}, \end{array}\text{\textbackslash{}]}$$

[3] $$\begin{array}{c} Repurchase\;likelihood_{ib}=\\ \alpha +\beta_{1}NoFarmDirectKnowledge_{i}+\beta_{2}FarmDirectPF_{i}\\ +\beta_{3}DairySpending_{i}+\theta Quality_{ib}+\beta_{4}LocalApproval_{i}+X_{i}\gamma \\ +\delta_{b}+\varepsilon_{ib}, \end{array}$$

[4] $$\text{\textbackslash{}[}\begin{array}{c} Recommendation\;likelihood=\\ \alpha +\beta_{1}NoFarmDirectKnowledge_{i}+\beta_{2}FarmDirectPF_{i}\\ +\beta_{3}DairySpending_{i}+\theta Quality_{ib}+\beta_{4}LocalApproval_{i}\\ +\beta_{5}RepurchaseLikelihood_{ib}+X_{i}\gamma +\delta_{b}+\varepsilon_{ib}, \end{array}\text{\textbackslash{}]}$$

where Aware_ib_ = respondent *i* knows brand *b* (1 = yes; 0 otherwise); NoFarmDirectKnowledge_i_ = 1 if respondent *i* lacks farm-direct knowledge at all, 0 otherwise; FarmDirectPF_i_ = annual farm-direct purchase frequency; DairySpending_i_ = monthly dairy spending for individual *i* (mean = 58.72, ranging from $20 to $200); LocalApproval_i_ = individual *i* approve of local foods; RepurchaseLikelihood_ib_ = individual *i*'s repurchase likelihood of brand *b*, ranging from 0 (0%, not at all likely) to 100 (100% likely); RecommendationLikelihood_ib_ = individual *i*'s repurchase likelihood of brand *b*, ranging from 0 (0%, not at all likely) to 100 (100% likely); Quality_ib_ = individual *i*'s perceived quality of brand *b* ranged 1 (poor quality) to 5 (high quality); X_i_ = demographic controls (i.e., age, gender, education, income); δ_b_ = brand fixed effects; and ε_ib_ = error terms.

Hypotheses were specified for each explanatory variable based on the literature review. It was hypothesized that no farm-direct knowledge would negatively affect awareness and purchase, because consumers without exposure have fewer opportunities to encounter local brands (Bradford and Durst, 2021). Second, it was hypothesized that farm-direct purchase frequency would positively affect all 4 stages of the marketing funnel (awareness, purchase, repurchase, and recommendation) because repeated engagement with direct channels informs consumer choice (Printezis et al., 2019). A small positive effect was expected for dairy spending across awareness and purchase. A positive effect was also expected for local food approval across all 4 marketing stages. This is consistent with evidence that consumers' attitudes toward local foods predict their purchase behavior (Cranfield et al., 2012). Quality was expected to be a dominant predictor of repurchase intent and recommendation, which is consistent with quality-driven loyalty models (Saleem et al., 2015; Esene, 2023). Finally, it was hypothesized that repurchase likelihood positively drives recommendations.

A total of 110 people participated in the study. Respondents were distributed across suburban (41.4%), urban (33.3%), rural (16.2%), and small-town (9.0%) settings (Table 1). The sample was predominantly female (63.9%), with a mean age of 35.9 years. A majority (73.0%) had attended university (4-year bachelor's degree or higher), and 77.5% self-identified as white or Caucasian, followed by Asian (10.8%), Black (4.5%), Hispanic (2.7%), and other remaining groups (4.5%). Mean household composition was 2 adults and 0.5 children. These demographic characteristics are broadly representative of engaged local food consumer samples reported in prior literature (Sanders, 2024).

**Table 1:** Definitions and summary of variables used in the study of consumer, awareness, purchase behavior, and brand loyalty in local and regional dairy market

Variable	Definition and coding	Mean	SD
Panel A: Demographics			
Age	Years	36.86	11.12
Male	1 = male, 0 = otherwise	0.351	0.478
University education	1 = bachelor's degree or higher, 0 = otherwise	0.730	0.444
White	1 = white, 0 = otherwise	0.775	0.418
Asian	1 = Asian, 0 = otherwise	0.108	0.311
Black	1 = Black, 0 = otherwise	0.045	0.208
Hispanic	1 = Hispanic, 0 = otherwise	0.027	0.162
Suburban	1 = suburban residence, 0 = otherwise	0.414	0.493
Urban	1 = urban residence, 0 = otherwise	0.333	0.472
Rural	1 = rural residence, 0 = otherwise	0.162	0.369
Small town	1 = small-town residence, 0 = otherwise	0.090	0.286
Income	Annual household income, $1,000s	81.22	54.53
Panel B: Dairy purchasing behavior			
Milk purchase frequency	Times/year	44.60	24.88
Cheese purchase	Times/year	40.83	23.37
Yogurt purchase frequency	Times/year	34.44	28.46
Ice cream purchase	Times/year	24.69	20.25
Monthly dairy spending	$/month	58.73	34.85
On-farm spending per visit	$/visit	41.98	23.68
Grocery store shopper	1 = uses grocery stores, 0 = otherwise	0.982	0.133
Farmers market shopper	1 = uses farmers markets, 0 = otherwise	0.225	0.418
Box store shopper	1 = uses box stores, 0 = otherwise	0.180	0.385
Mass merchandiser shopper	1 = uses mass merchandisers, 0 = otherwise	0.171	0.377
Specialty store shopper	1 = uses specialty stores, 0 = otherwise	0.117	0.322
Co-op shopper	1 = uses co-ops, 0 = otherwise	0.081	0.273
Online shopper	1 = uses online retailers, 0 = otherwise	0.045	0.208
Farmstand shopper	1 = uses farmstands, 0 = otherwise	0.027	0.162
Panel C: Regression model variables			
Quality	1 (poor) to 5 (high) scale, conditional on purchase	4.41	0.80
NoFarmDirectKnowledge	1 = lowest self-rated farm-direct knowledge, 0 = otherwise	0.270	0.444
FarmDirectPF	Annual farm-direct purchase frequency	2.90	6.45
LocalApproval	1 = agree/strongly agree approve buying local foods, 0 = otherwise	0.964	0.186

Grocery stores were the retail channel for most respondents (99%), followed by farmers markets (22.5%), box stores (18%), mass merchandisers (17%), specialty stores (13%), co-ops (8%), online retailers (5%), and farmstands (4%). The above-average farmers market participation reflects a sample with potentially above-average local food orientations, consistent with prior investigations that farmers market shoppers exhibit stronger local food preferences and greater familiarity with local brands (Duke, 2025).

The marketing funnel results for all 7 dairy brands (Table 2) reveal substantial heterogeneity across all funnel stages. The regional brand achieved the highest brand awareness, at 93.7%. Local brand A was second at 85.0%, followed by LB at 63.1%. The 3 smaller local producers, LC, LD, and LE, all registered awareness below 10% significance, highlighting a significant gap between established and small local creameries. Notably, 4.5% of respondents reported awareness of the hypothetical brand, reflecting a low to modest yea-saying rate, consistent with yea-saying bias (Jayanti et al., 2004), where participants report recognition or awareness of product brands whether they are actually aware or not. Considering the magnitude of the bias and our sample size, we retained all respondents for our analysis. However, it should be noted that the 3 brands with low awareness were located in Middle Tennessee, and data collection occurred in East Tennessee, so brand awareness may be suppressed given the study location. Future work could use the same metrics and brands but using a Middle Tennessee sample to test the robustness of local brands versus more available brands.

**Table 2:** Mean ratings of the 5 marketing funnel metrics recorded for 6 real and 1 hypothetical dairy brands available in Tennessee in 2024 (n = 110)

Brand	Awareness,^1^ % (SD)	Purchase,^2^ % (SD)	Repurchase,^3^ mean (SD)	Recommendation,^3^ mean (SD)	Quality,^4^ mean (SD)
Regional brand	93.69 (24.41)	92.30 (26.7)	89.18 (19.13)	79.80 (24.20)	4.05 (0.89)
Local brand A	84.68 (36.17)	90.40 (29.58)	91.09 (18.24)	91.26 (16.85)	4.66 (0.66)
Local brand B	63.06 (48.48)	74.30 (44.02)	91.82 (15.38)	90.85 (18.39)	4.64 (0.62)
Local brand C	9.90 (30.00)	54.50 (52.22)	89.50 (20.04)	85.00 (19.75)	4.50 (0.84)
Local brand D	8.14 (27.00)	54.50 (52)	90.00 (24.49)	90.00 (20.00)	4.33 (0.82)
Local brand E	9.90 (30.00)	33.30 (5)	95.00 (10.00)	98.75 (2.50)	4.75 (0.50)
Hypothetical brand (fabricated)	4.46 (20.30)	0.00	—	—	—

1 Brand awareness indicates the portion of all respondents (n = 110) recognizing the brand (1 = aware, 0 otherwise).

2 Purchase indicates portion of the aware respondents reporting prior purchase (1 = purchased, 0 otherwise).

3 Repurchase and recommendation are mean scores on likelihood scales from 0 to 100, conditional on prior purchases of each brand.

4 Quality was measured by the statement, “Please indicate your perception of the product quality (1 = poor; 5 = high quality).”

Conditional on awareness, purchase conversion was high for established brands: 92.3% for the regional brand and 90.4% for LA. In LB, 74.3% of aware consumers were purchasers, and the smaller local brands exhibited lower rates, including 54.5% for LC and LD, and 33.3% for the LE. None of the hypothetical brand-aware respondents reported purchasing the brand, confirming the validity of the purchase measure. Repurchase likelihood was high across all brands, led by LE (95% repurchase likelihood) and LB (92%), which both exceeded the regional brand (79.8%). Recommendation likelihood scores showed an even more pronounced local brand advantage, led by LE (98.8%), then LA (91.3%), LB (91%), and LD (90%). All of the local brands substantially exceeded the regional brand's recommendation likelihood score (79.8%), suggesting that local brand purchasers are potentially enthusiastic brand advocates.

Table 3 reports model estimates. For model 1, brand awareness (R^2^ = 0.59), after controlling for consumer characteristics, all brands showed significantly lower awareness levels than the regional brand. The LC, LD, and LE registered lower awareness, exceeding 83 percentage points. The hypothetical brand registered the largest negative coefficient, as expected. Among consumer-level predictors, approval of buying local foods had a positive, significant effect on awareness (*P* = 0.001), and absence of farm-direct knowledge significantly reduced awareness (*P* = 0.001). Conventional demographic variables were not significant predictors of brand awareness at 10% and were suppressed in the models presented. Model 2 addressed purchase likelihood (R^2^ = 0.58). After controlling for demographics, product perceptions, information, and preferences, the 5 local brands (LA, LB, LC, LD, and LE) exhibited lower purchase probabilities than the regional brand. Farm-direct purchase frequency (*P* = 0.067) and local food buying approval both significantly increased purchase likelihood (*P* < 0.001). Model 3 addressed repurchase likelihood (R^2^ = 0.338), which shows estimates among respondents who have purchased a branded product. Perceived product quality was the dominant determinant of repurchase intention. It exhibited the largest effect in the model, which is consistent with the food quality loyalty literature (Saleem et al., 2015; Kumar et al., 2025). Model 4 reports determinants of recommendation likelihood (R^2^ = 0.364). As in the repurchase model, perceived product quality is a strong predictor of recommendation intentions. Repurchase likelihood also plays a central role in driving recommendations, with a highly significant coefficient (*P* < 0.001), consistent with the notion that repeat purchase precedes consumer advocacy. Brand effects in the recommendation model indicate that several local brands generate higher recommendation likelihood ratings relative to the regional brand, including LA, LB, LC, and LD. The brand effect of LC was not statistically significant relative to the regional brand (*P* = 0.58). Among consumer-level predictors, the farm-direct purchase frequency positively affects recommendation likelihood, suggesting that consumers more engaged with local food systems are more likely to recommend these local dairy brands.

**Table 3:** Tennessee consumer decision funnel estimates for dairy brands: Awareness, purchase, repurchase, and recommendation (n = 110; Coef. = coefficient)

Model^1^	(1) Awareness		(2) Purchase		(3) Repurchase		(4) Recommend	
	Coef. (SE)	*P*-value	Coef. (SE)	*P*-value	Coef. (SE)	*P*-value	Coef. (SE)	*P*-value
Brand								
Local brand A	−0.091** (0.038)	0.019	−0.100** (0.049)	0.043	−4.851* (2.503)	0.055	5.988*** (1.621)	<0.001
Local brand B	−0.309*** (0.045)	<0.001	−0.364*** (0.054)	<0.001	−5.51** (2.465)	0.039	4.799*** (1.7835)	0.008
Local brand C	−0.836*** (0.038)	<0.001	−0.809*** (0.038)	<0.001	−7.812 (9.075)	0.392	1.204 (2.352)	0.51
Local brand D	−0.836*** (0.040)	<0.001	−0.809*** (0.042)	<0.001	−0.129 (3.556)	0.971	7.047*** (1.838)	<0.001
Local brand E	−0.855*** (0.034)	<0.001	−0.827*** (0.037)	<0.001	−0.549 (4.653)	0.907	6.43*** (2.381)	0.008
Hypothetical brand	−0.891*** (0.030)	<0.001	−0.864*** (0.033)	<0.0010	—		—	
Information and preferences								
No farm-direct knowledge	−0.101*** (0.030)	0.001	−0.067** (0.030)	0.025	−6.296** (2.849)	0.029	1.571 (2.169)	0.471
Farm-direct purchase frequency	0.001 (0.001)	0.402	0.003* (0.002)	0.067	0.003 (0.121)	0.807	0.224*** (0.085)	0.010
Monthly dairy spending	−0.001 (0.001)	0.321	−0.000 (0.001)	0.609	−0.080** (0.037)	0.031	0.005 (0.022)	0.809
Approve buying local	0.166*** (0.050)	0.001	0.159*** (0.041)	<0.001	3.970 (14.583)	0.786	−2.896 (3.446)	0.403
Product perceptions								
Perceived quality	—		—		11.733*** (2.050)	<0.001	7.885*** (1.330)	<0.001
Repurchase likelihood	—		—		—		0.744*** (0.053)	<0.001
Demographic controls^2^	Yes		Yes		Yes		Yes	
Observations	770		770		250		249	
R^2^	0.596		0.58		0.358		0.764	

1 Regional brand is the reference variable. Local brands C, D, and E had fewer than 10 purchasers each, so their brand effect should be considered only descriptive rather than inferential.

2 Demographic controls included residency, gender, race or ethnicity, income, education, family size (adult and children), and age. The models including the demographic controls are available from the corresponding author upon request.

* *P* < 0.1; **P < 0.05; ***P < 0.01.

The findings highlight a significant awareness gap between the established brand (regional brand; 93.69%) and the local brands. Proximity greatly influences awareness with the local brands in the study area exhibiting stronger awareness (62.5% to 84.7%), then the local creameries located further away (8% to 10%), a difference of up to 76.7 percentage points. Although the dominant regional brand exhibited near universal awareness, awareness of local dairy brands remained limited. Local dairy brands have potential to expand their customer base through generating awareness. This aligns with the literature that argues availability and awareness are major constraints facing small and regional food producers (Rubio et al., 2014). Consumers who report approval of buying local foods and familiarity with farm-direct purchasing channels are more likely to recognize local dairy brands. This supports the argument that farmers markets, farmstands, and other direct marketing channels function not only as retail outlets but also as social and informational networks through which brand familiarity is built (Trachuk et al., 2021). The absence of significant demographic effects suggests that awareness disparities arise mainly from differences in exposure than from demographic attributes. The purchase stage reinforces the role of consumer engagement with local food systems in shaping marketing outcomes for local dairy brands. Individuals who participate in farm-direct environments are more likely to purchase local dairy brands.

Once consumers have purchased a product, perceived high quality appears to be the central factor shaping subsequent loyalty behaviors (i.e., repurchase and recommendation likelihood). This relationship is consistent with established models of consumer loyalty where satisfaction drives repeat purchase and positive word of mouth (Hart et al., 2007; Chatzoglou et al., 2022). Importantly, quality perceptions were measured only among consumers who have previously purchased the product. The findings therefore reflect post-purchase evaluations. In the context of food products, consumers typically learn about product quality through consumption than through observable attributes before purchase. Retail scanner data could be used to identify the robustness of this observation using real-world purchasing data.

The analyses also highlight the sequential structure of consumer loyalty formation. Repurchase intentions are strongly associated with recommendation behavior, indicating that consumers who expect to buy a product again are more likely to advocate for that product. Positive consumption experiences transform occasional buyers into repeat customers and informal brand promoters.

Together, the results suggest a 2-stage challenge for local dairy producers. The first challenge lies in overcoming substantial awareness gaps that separate smaller brands from dominant commercial competitors. Without initial brand recognition, potential consumers may never reach the stage at which product quality influences loyalty. The second challenge concerns the maintenance of product quality once consumers make the initial purchase. Among existing buyers, perceived quality strongly shapes repurchase and recommendation behavior. This implies that, although marketing investments may help initial awareness, sustained market success ultimately depends on delivering a product experience that meets consumer expectations. These findings underscore the complementary roles of information exposure and product performance for local dairy brands. Direct marketing channels, community engagement, and other visibility-enhancing strategies may help in first purchase decisions, and product quality determines whether those consumers remain loyal and advocate for the brand.

Several limitations should be noted when interpreting these findings. The cross-sectional nature of the design prevents causal inference between attitudes (local food approval) and behavior (purchase). Second, the sample of 110 Tennesseans limits the statistical power, and the relatively high university rate (73%) and farmers market participation (22.5%) suggest positive selection toward local food engagement, limiting generalizability. Third, self-reported measures are subject to biases such as recall and social desirability bias. Finally, the brands and market are specific to the Tennessee dairy context. Future research could build upon these findings by expanding the geographic range and incorporating larger, national brands.

## References

[bib1] Bajgain P., Rihn A., Eckemkamp L., Martinez C. (2026). Media matters: Rich media (video) vs. infographics on consumer valuation of cheese attributes. J. Agric. Food Res..

[bib2] Bradford B., Durst P. (2021). Consumers Desire Local Dairy Products.. https://www.canr.msu.edu/news/consumers-desire-local-dairy-products.

[bib3] Cameron A.C., Miller D.L. (2015). A practitioner's guide to cluster-robust inference. J. Hum. Resour..

[bib4] Chatzoglou P., Chatzoudes D., Savvidou A., Fotiadis T., Delias P. (2022). Factors affecting repurchase intentions in retail shopping: An empirical study. Heliyon.

[bib5] Coelho F.C., Coelho E.M., Egerer M. (2018). Local food: Benefits and failings due to modern agriculture. Sci. Agric..

[bib6] Cranfield J., Henson S., Blandon J. (2012). The effect of attitudinal and sociodemographic factors on the likelihood of buying locally produced food. Agribusiness.

[bib7] de Haan E., Wiesel T., Pauwels K. (2016). The effectiveness of different forms of online advertising for purchase conversion in a multiple-channel attribution framework. Int. J. Res. Mark..

[bib8] DeLind L.B. (2011). Are local food and the local food movement taking us where we want to go? Or are we hitching our wagons to the wrong stars?. Agric. Human Values.

[bib9] Duke S. (2025). https://www.psu.edu/news/agricultural-sciences/story/penn-state-extension-survey-reveals-strengths-growth-areas-farmers.

[bib10] Esene F. (2023).

[bib11] Hart C., Farrell A.M., Stachow G., Reed G., Cadogan J.W. (2007). Enjoyment of the shopping experience: Impact on customers' repatronage intentions and gender influence. Serv. Ind. J..

[bib12] Hogan J.E., Lemon K.N., Libai B. (2004). Quantifying the ripple: Word-of-mouth and advertising effectiveness. J. Advert. Res..

[bib13] IDFA (2021). US dairy industry's economic impact totals $753 billion.. https://www.idfa.org/news/u-s-dairy-industrys-economic-impact-totals-753-billion.

[bib14] Jayanti R.K., McManamon M.K., Whipple T.W. (2004). The effects of aging on brand attitude measurement. J. Consum. Mark..

[bib15] Kristensen T., Gabrielsen G., Zaichkowsky J.L. (2012). How valuable is a well-crafted design and name brand? Recognition and willingness to pay. J. Consum. Behav..

[bib16] Kumar R., Jain V., Eastman J.K., Ambika A. (2025). The components of perceived quality and their influence on online re-purchase intention. J. Consum. Mark..

[bib17] Lemon K.N., Verhoef P.C. (2016). Understanding customer experience throughout the customer journey. J. Mark..

[bib18] Li H., Kannan P.K. (2014). Attributing conversions in a multichannel online marketing environment: An empirical model and a field experiment. J. Mark. Res..

[bib19] MacDonald J.M., Law J., Mosheim R. (2020). https://ers.usda.gov/sites/default/files/_laserfiche/publications/98901/ERR-274.pdf?v=11513.

[bib20] Printezis I., Grebitus C. (2018). Marketing channels for local food. Ecol. Econ..

[bib21] Printezis I., Grebitus C., Hirsch S. (2019). The price is right!? A meta-regression analysis on willingness to pay for local food. PLoS One.

[bib22] Rossi J., Woods T. (2024). How do residents perceive local food system activity? Perspectives of system performance and awareness using the local food vitality survey. Local Dev. Soc..

[bib23] Rubio N., Oubiña J., Villaseñor N. (2014). Brand awareness—Brand quality inference and consumer's risk perception in store brands of food products. Food Qual. Prefer..

[bib24] Saleem A., Ghafar A., Ibrahim M., Yousuf M., Ahmed N., Ahmad S.F. (2015). Product perceived quality and purchase intention with consumer satisfaction. Global J. Manag. Bus. Res..

[bib25] Sanders K. (2024). https://content.ces.ncsu.edu/overview-of-local-food-trends-recommendations-for-communication-and-marketing.

[bib26] Trachuk T., Vdovichena O., Andriushchenko M., Semenda O., Pashkevych M. (2021). Branding and advertising on social networks: current trends. Int. J. Comput. Sci. Netw. Secur..

[bib27] Varziri A., Zhao S., Woods T.A., Esene F. (2025). Consumer preferences toward local versus carbon: A study of value-added dairy products. Agric. Econ..

[bib28] Zaring C., Rihn A., Eckelkamp L. (2024). https://utia.tennessee.edu/publications/wp-content/uploads/sites/269/2024/12/W1286.pdf.

[bib29] Zepeda L., Li J. (2006). Who buys local food?. J. Food Distrib. Res..

